# Investigating the implant position reproducibility of optical impressions obtained using an intraoral scanner and 3D-printed models fabricated using an intraoral scanner

**DOI:** 10.1186/s40729-023-00481-3

**Published:** 2023-06-21

**Authors:** Maya Iwamoto, Wataru Atsuta, Yasuhide Kaneko, Junnosuke Ito, Takeshi Kanno, Takahiro Murakami, Jyoji Tanaka

**Affiliations:** Clinical Implant Society of Japan, 1-43-9, Komagome, Toshima-ku, Tokyo, 170-0003 Japan

**Keywords:** Full-arch implant cases, Intraoral scanner, 3D printer, Plaster model, Implant position reproducibility

## Abstract

**Purpose:**

This study aims to examine the effect of the size of the intraoral scanning area on implant position reproducibility and compare the implant position reproducibility of plaster models fabricated using the silicone impression technique, the digital model of an intraoral scanner, and three-dimensional (3D)-printed models fabricated using an intraoral scanner.

**Methods:**

Scanbodies were attached to an edentulous model with six implants (master model) and were scanned using a dental laboratory scanner to obtain basic data. The plaster model was fabricated using the open-tray method (IMPM; *n* = 5). The master model was then scanned in various implant areas using an intraoral scanner to obtain data (IOSM; *n* = 5); the scanning data of six scanbodies were used to fabricate the 3D-printed models (3DPM; *n* = 5) using a 3D printer. Scanbodies were attached to the implant analogs of the IMPM and 3DPM models and data were obtained using a dental laboratory scanner. The basic data and IMPM, IOSM, and 3DPM data were superimposed to calculate the concordance rate of the scanbodies.

**Results:**

The concordance rate of intraoral scanning decreased as the number of scanbodies increased. Significant differences were observed between the IMPM and IOSM data, and between the IOSM and 3DPM data; however, the IMPM and 3DPM data did not differ significantly.

**Conclusions:**

The implant position reproducibility of the intraoral scanner decreased with an increase in the scanning area. However, ISOM and 3DPM may provide higher implant position reproducibility than plaster models fabricated using IMPM.

## Background

Digital dental technology has progressed significantly, and thus, it has been used for various applications ranging from the fabrication of surgical guides for implant placement and fixed dental prostheses to aligner orthodontics [1–6]. The application of these digital technologies in clinical dentistry can improve the accuracy of diagnosis and treatment while simultaneously improving patients’ quality of life [7–11].

Among the digital dental technologies, dental computer-aided design/computer-aided manufacturing (CAD/CAM) systems have progressed significantly. In the 1990s, the average error in dental CAD/CAM systems was reported to be 200 μm [12]; however, the current dental laboratory scanners (D1000, 3Shape, Copenhagen, Denmark) have a high average precision of 0.5 μm, suggesting that their accuracy is comparable to that of the high-precision industrial scanners [13]. In addition to dental laboratory scanners, intraoral scanners have exhibited excellent performance [13–15]. Murakami et al. placed implants in a jaw model with a single missing tooth and compared the implant position reproducibility obtained by intraoral scanners and the silicone impression technique; they obtained better results with the former than with the latter [16]. Pesce et al. placed four implants in an edentulous maxillary model and scanned them using an intraoral scanner to fabricate a metal framework, which showed a good fit [17]. Three-dimensional (3D) printers also possess high precision level, which is comparable to that of intraoral scanners. Several researchers have reported that the use of 3D-printed surgical guides results in accurate implant placement and yields excellent clinical results [18–20]. Tanaka et al. compared the accuracy of implant-supported copy overdentures fabricated using an intraoral scanner and a 3D printer with that of copy overdentures fabricated using conventional impression materials and room-temperature curing resin; they found that the accuracy of the former was significantly higher than that of the latter, confirming the high accuracy of intraoral scanners and 3D printers [21, 22]. However, the effect of an increase in the number of implants on the accuracy of impression in the optical impression technique using an intraoral scanner, the implant position reproducibility of intraoral scanners when they are applied to full-arch implant, as well as working models fabricated using an intraoral scanner combined with a 3D printer, have not been investigated in detail.

This study aimed to examine the effect of the size of the intraoral scanning area on implant position reproducibility and compare the implant position reproducibility of plaster models fabricated using the silicone impression technique with that of optical impressions obtained using an intraoral scanner and 3D-printed models obtained using an intraoral scanner. Herein, six implants were placed in an edentulous maxillary plaster model, and the area of each implant was scanned using an intraoral scanner to determine the appropriate number of implants. Subsequently, the working models were fabricated using the conventional silicone impression technique and a combination of an intraoral scanner and a 3D printer. The implant position reproducibility of all the models were then compared. The null hypothesis of this study was that the size of the intraoral scanning area would not affect the implant position reproducibility and that the implant position reproducibility obtained by plaster models fabricated using the silicone impression technique and 3D-printed models fabricated using an intraoral scanner will not differ significantly.

## Materials and methods

### Fabrication of the master model and acquisition of basic data

**Fig. 1:**
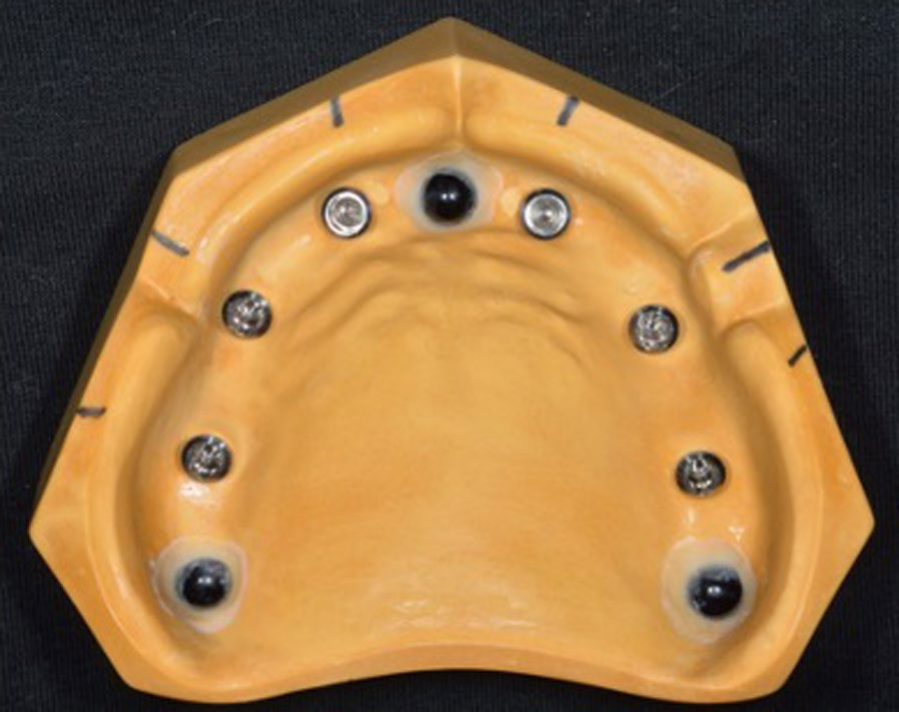
Master model

To fabricate a master model, six implants (Roxolid Tissue Level Standard Implant Ø 4.1 mm RN-SLActive Loxim-8 mm, Straumann, Basel, Switzerland) were placed in different parts of an edentulous maxillary plaster model corresponding to tooth numbers 16, 14, 12, 22, 24, and 26. Implants corresponding to teeth 16, 14, 24, and 26 were placed perpendicular to the virtual occlusal plane, whereas those corresponding to teeth 12 and 22 were placed labially inclined. The insertion angles of the implants were measured using a surveyor. Reference bodies were then placed between teeth 12 and 22 and behind teeth 16 and 26 (Fig. 1).

Thereafter, scanbodies (CARES Mono Scanbody RN, Straumann) were attached to the implants on the master model and scanned to obtain the basic master model data using a dental laboratory scanner (3Shape) with the accuracy of 5 μm [ISO 12836]. The basic data obtained were saved as a standard triangulated language (STL) file.

### Fabrication of working models using the silicone impression technique and acquisition of the data

**Fig. 2:**
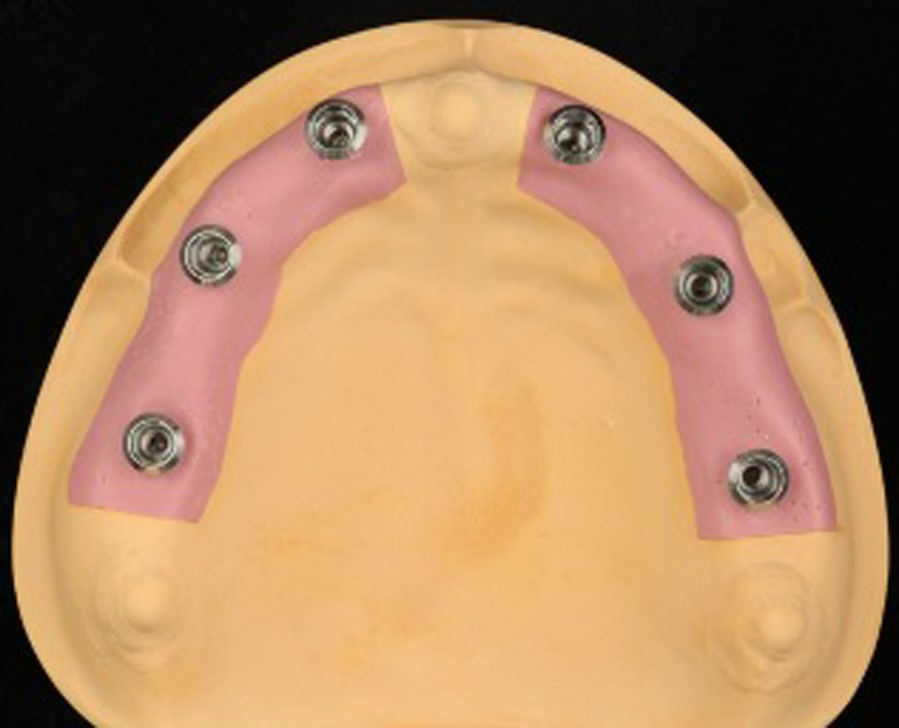
Working model fabricated using the silicone impression technique

An individual tray for the master model was prepared to obtain precise impression of the implants. Impression copings (RN synOcta Impression Cap, Straumann) were attached to the implants on the master model, and a precise impression of the implants was obtained using a silicone impression material (Imprint™ 4, 3M, St Paul, USA). Attachment of the impression copings to the implants in a consistent state was performed by a dentist with 10 years postgraduate experience, using the tightening manual recommended by the manufacturer. After removing the impression material, the implant analog (RN synOcta Implant Analog, Straumann) was attached to the impression copings inside the impression, and plaster was poured onto the impression to fabricate the working models (impression working model [IMPM], *n* = 5) (Fig. 2). Scanbodies were then attached to the implant analogs on the working models, which were stored in a desiccator for 1 week and scanned using a dental laboratory scanner to obtain the scanning data. The scanning data were saved as STL files.

### Acquisition of the master model data using an intraoral scanner

**Fig. 3:**
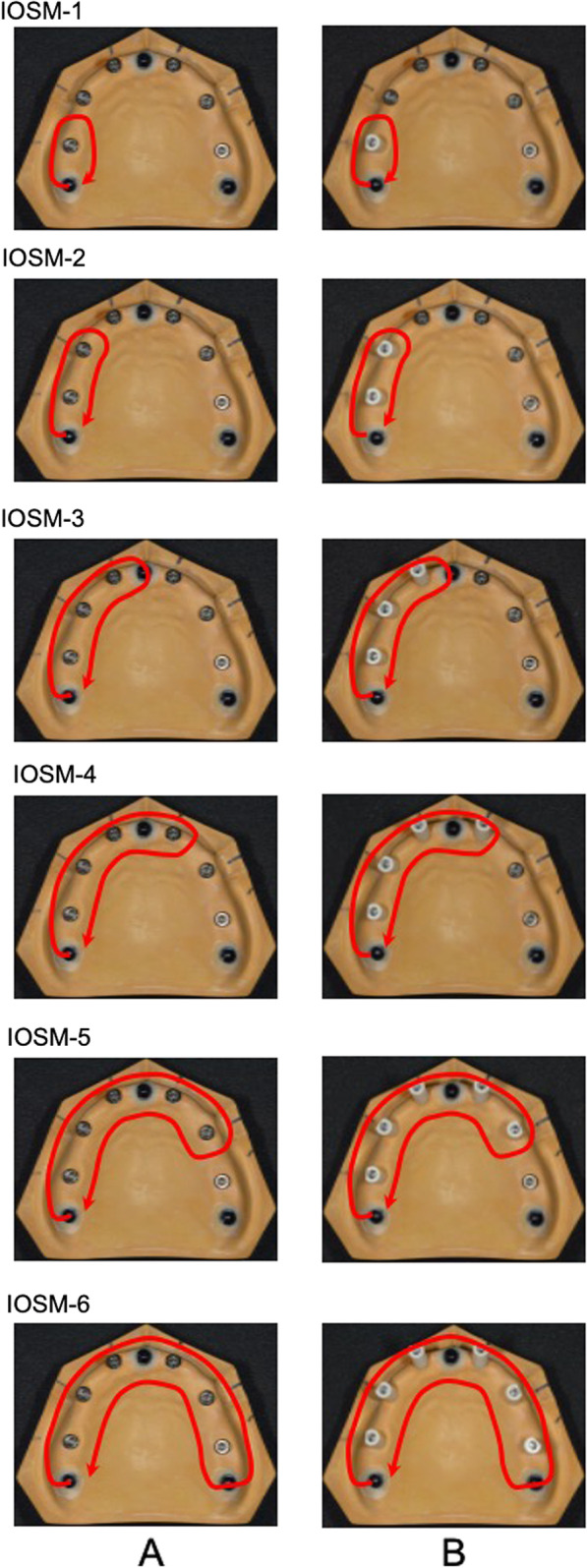
Scanning of the **A** alveolar ridge and **B** scanbodies

Optical impressions of the master model with varying numbers of implants from one to six were obtained using an intraoral scanner (Trios 3, 3Shape, Copenhagen, Denmark). First, the alveolar ridge of the master model was scanned (Fig. 3A). Scanbodies were then attached to the implants and scanned in full view, as shown in Fig. 3B (IOSMs) [IOSM-1 (scanning of a scanbody corresponding to tooth 16), IOSM-2 (scanning of scanbodies corresponding to teeth 16 and 14), IOSM-3 (scanning of scanbodies corresponding to teeth 16, 14, and 12), IOSM-4 (scanning of scanbodies corresponding to teeth 16, 14, 12, and 22), IOSM-5 (scanning of scanbodies corresponding to teeth 16, 14, 12, 22, and 24), IOSM-6 (scanning of scanbodies corresponding to teeth 16, 14, 12, 22, 24, and 26)], *n* = 5). Attachment of the scanbodies to the implants in a constant state was also performed by the same operator, using the tightening manual recommended by the manufacturer. The scanning data obtained were saved as STL files.

### Fabrication of working models using an intraoral scanner and a 3D printer and acquisition of the data

**Fig. 4:**
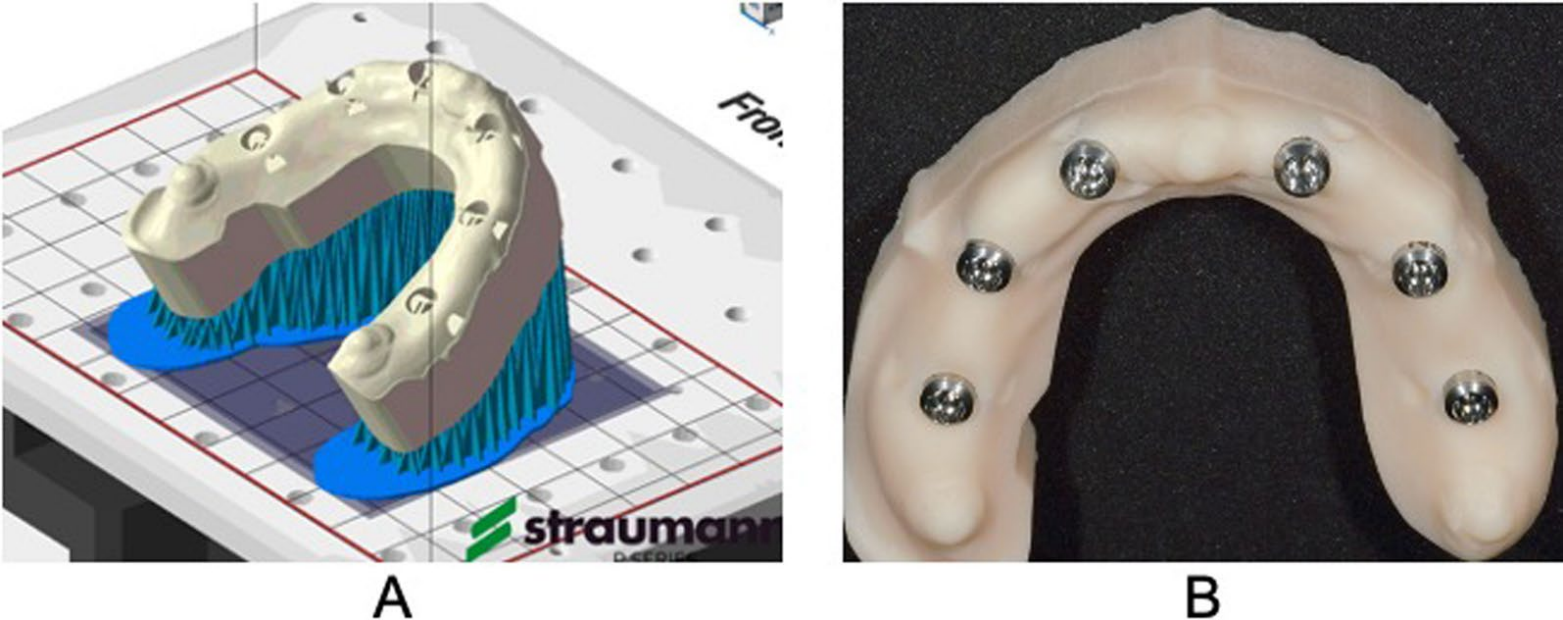
**A** Data used for printing of the working models, and **B** working model fabricated using an intraoral scanner and a 3D printer

The master model data acquired using an intraoral scanner (IOSM-6) were fed to CARES® Visual 8.0 software (Straumann) and then imported into a 3D printer (P30, Straumann) to fabricate the working models using the printing material (FREEPRINT temp, DETAX GmbH & Co. KG, Ettlingen, Germany). The forming angle of the working model was set to 45° (Fig. 4A), and the lamination pitch was set to 100 μm. After the formation was complete, the working models were ultrasonically washed with 100% isopropyl alcohol for 5 min, dried at 25 °C for 30 min, and photoflashed 2000 times with a photopolymerization system (Flash-light plus, SHERA Werkstoff Technologie, GmbH & Co. KG, Lemförde, Germany) for final curing. Subsequently, to complete the working models, the implant analogs for the digital models (RN Implant Analog [Digital], Straumann) were placed in the parts of the working models corresponding to teeth 16, 14, 12, 22, 24, and 26 (Fig. 4B).

To obtain the data for the working models fabricated using a 3D printer, scanbodies were attached to the implant analogs on the working models, which were then stored in a desiccator for 1 week and scanned using a dental laboratory scanner (3D-printed model [3DPM], *n* = 5). The obtained basic data were saved as STL files.

### Data analysis

**Fig. 5:**
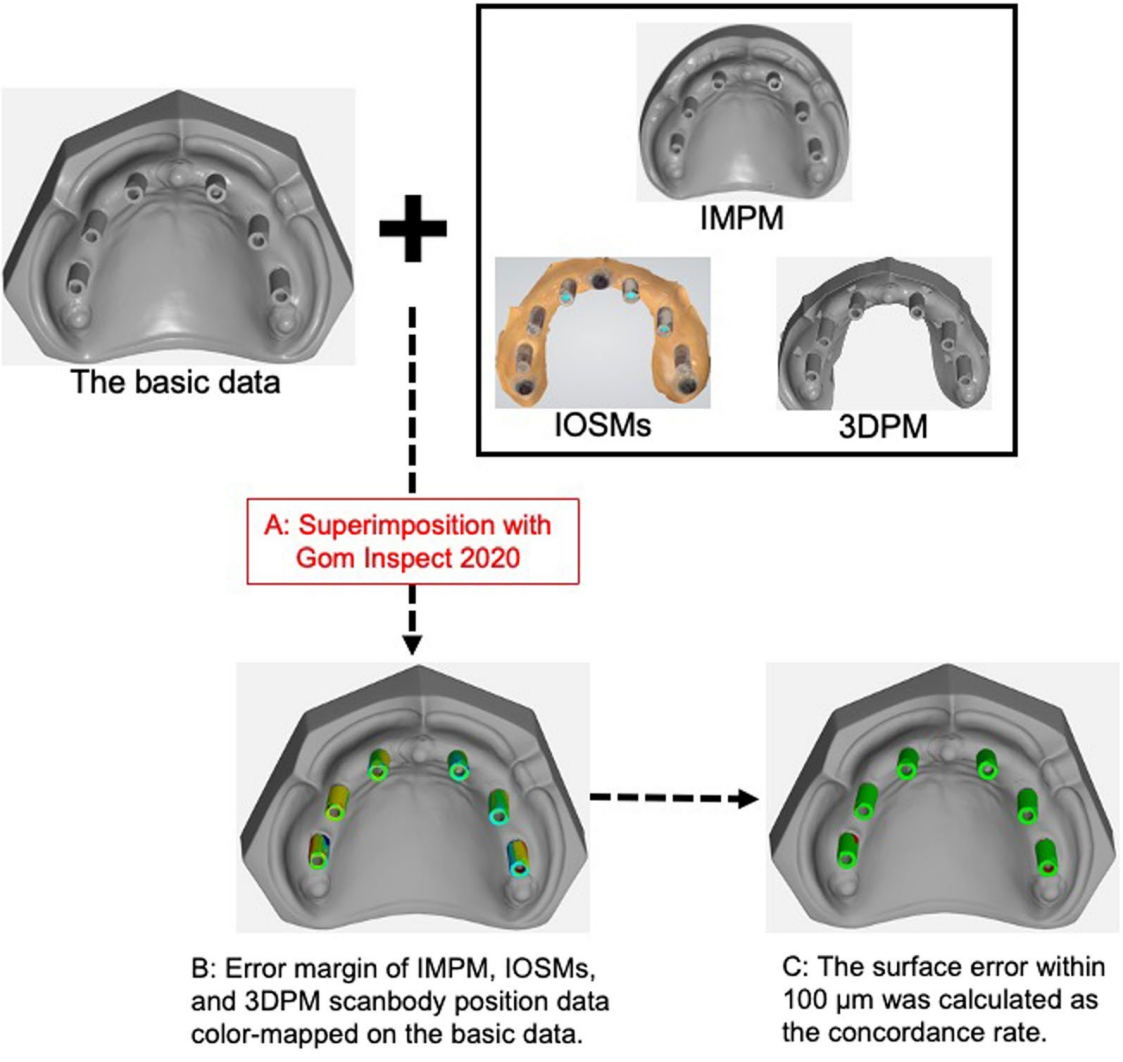
Calculation procedure of the color mapping image and concordance rate

The basic data, IMPM, IOSMs, and 3DPM data were imported into the 3D analysis software (Gom Inspect 2020, GOM GMBH, Braunschweig, Germany) to superimpose all the model data with the basic data; the three reference bodies were used as the reference points for superimposition (Fig. 5A).

To calculate the concordance rate of the scanbody to the surface area for each model, the position data of the scanbodies in IMPM, IOSMs, and 3DPM were color-mapped on the basic data of the scanbodies (Fig. 5B), and the error margin in the superstructure position was 100 μm or less, which was considered concordant [23–25] (Fig. 5C).

### Statistical processing

In this study, the sample size was determined with reference to the published articles [26–28]. The median and interquartile range (IQR) of the mean concordance rates of the scanbodies were determined for IMPM, IOSMs, and 3DPM. These values were statistically analyzed using the Kruskal–Wallis test followed by the Steel–Dwass multiple comparison test to determine the significant differences at a significance level of 5%.

## Results

### Concordance rate of each scanbody area by optical impression technique using an intraoral scanner

**Fig. 6:**
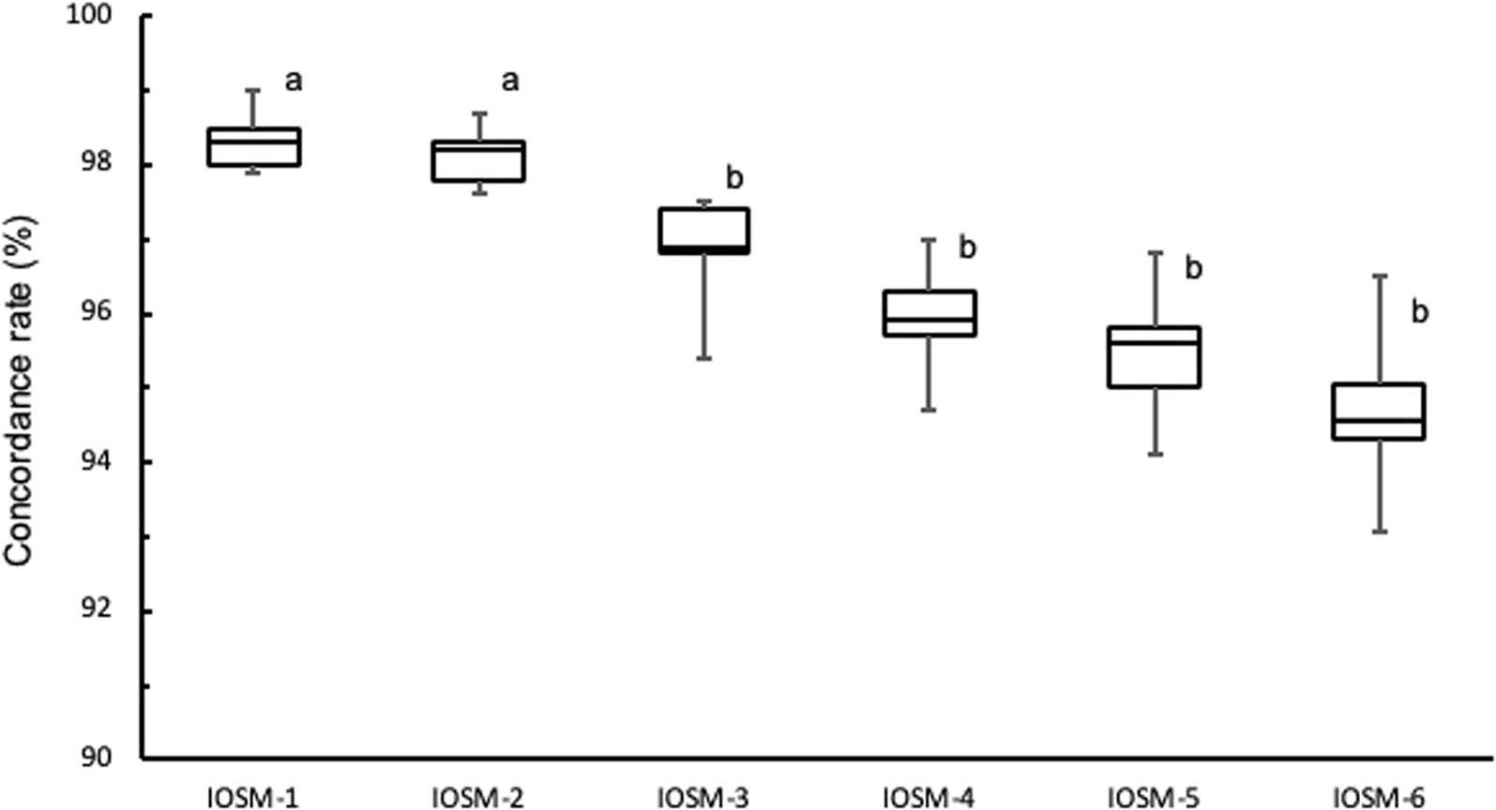
Concordance rate of each scanbody area by the optical impression technique using an intraoral scanner. Significant differences were observed between the median concordance rates “a” and “b” (*p* < 0.05)

The scanbody concordance rates based on the number of scanbodies are shown in Fig. 6. The median concordance rate (interquartile range) was 98.3% (0.5) for IOSM-1, 98.2% (0.5) for IOSM-2, 96.9% (0.6) for IOSM-3, 95.9% (0.6) for IOSM-4, 95.6% (0.8) for IOSM-5, and 94.6% (0.8) for IOSM-6. The median rate was the highest for IOSM-1, followed by IOSM-2, IOSM-3, IOSM-4, IOSM-5, and IOSM-6. Statistical processing showed significant differences between IOSM-1 and IOSM-3, IOSM-4, IOSM-5, and IOSM-6, and between IOSM-2 and IOSM-3, IOSM-4, IOSM-5, and IOSM-6 (*p* < 0.05); however, not between IOSM-1 and IOSM-2 (*p* > 0.05).

### Mean concordance rates of six scanbodies of working models fabricated through the silicone impression technique, digital models obtained by optical impressions taken with an intraoral scanner, and working models fabricated using an intraoral scanner and a 3D printer

**Fig. 7:**
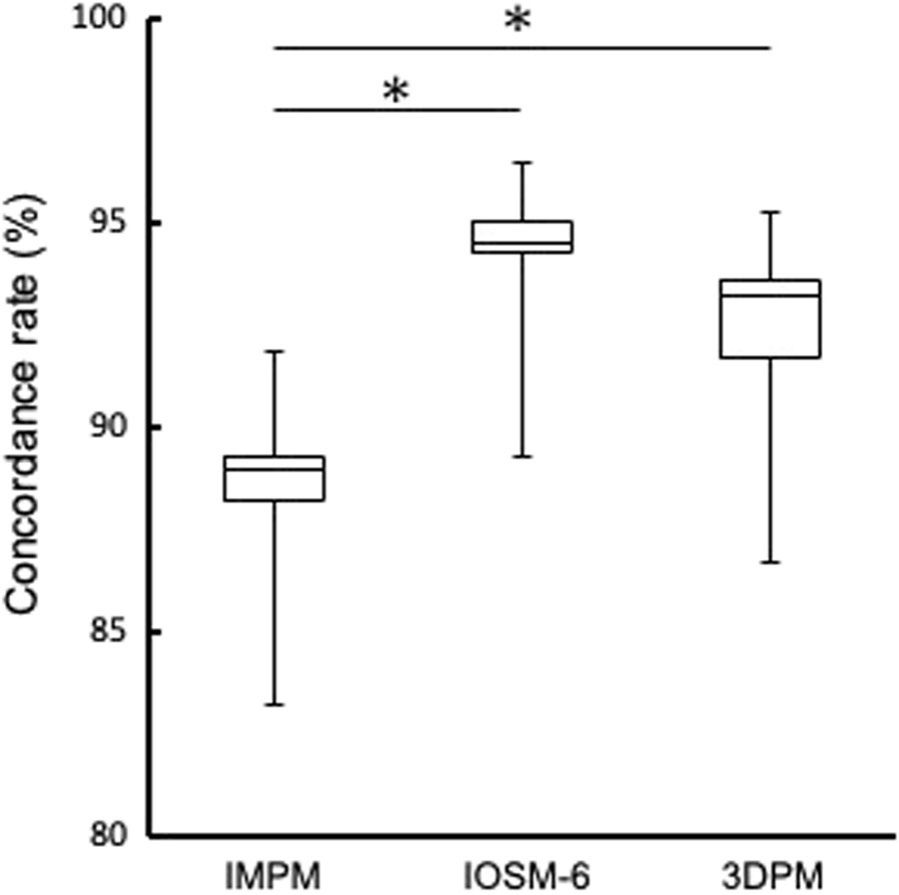
Mean concordance rates of the six scanbodies of the working models fabricated using the silicone impression technique (IMPM), digital models obtained by optical impressions taken using an intraoral scanner (IOSM-6), and working models fabricated using an intraoral scanner and a 3D printer (3DPM). Significant differences were observed between IMPM and IOSM-6 and between IOSM-6 and 3DPM (*p* < 0.05)

The mean concordance rates of the six scanbodies are shown in Fig. 7. The median (IQR) concordance rates were 89.0% (1.1) for IMPM, 94.6% (0.8) for IOSM-6, and 93.3% (1.8) for 3DPM. The median value was highest in IOSM-6, followed by that in 3DPM and IMPM. Significant differences were observed between the values in IMPM and IOSM-6 and between those in IMPM and 3DPM (*p* < 0.05); however, the median values in IOSM-6 and 3DPM (*p* > 0.05) did not differ significantly.

### Comparison of scanbody concordance rate by tooth position

**Fig. 8:**
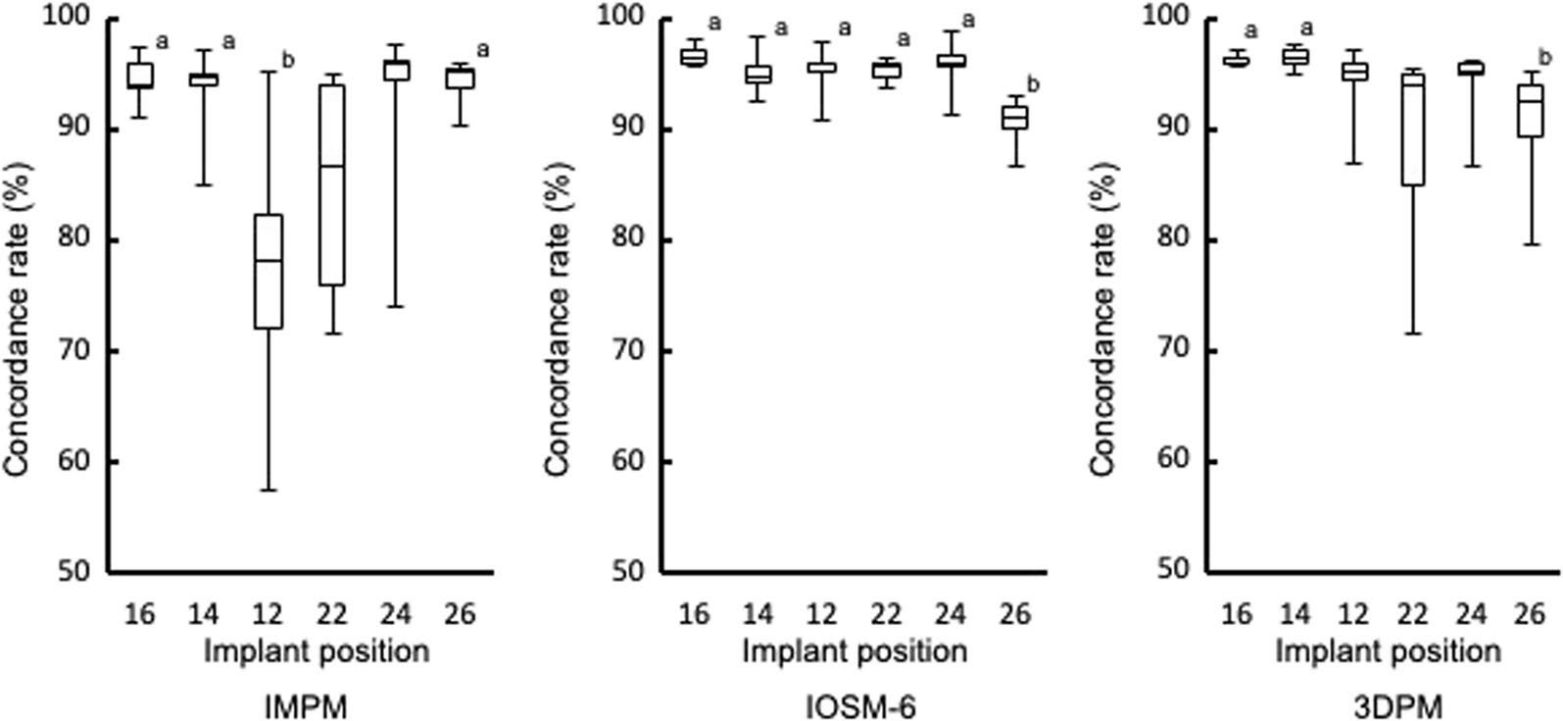
Concordance rates of scanbodies at each position. Significant differences were observed between the median concordance rates “a” and “b” (*p* < 0.05)

The scanbody concordance rates by tooth position are shown in Fig. 8.

The median (IQR) scanbody concordance rates at each tooth position (16, 14, 12, 22, 24, and 26, respectively) were as follows: 94.0% (2.2), 94.8% (1.1), 78.2% (10.1), 86.6% (18.0), 95.8% (1.6), and 95.1% (1.7) for IMPM; 96.3% (1.0), 94.7% (1.4), 95.8% (0.6), 95.7% (1.3), 95.7% (1.1), and 91.0% (1.9) for IOSM-6; and 96.0% (0.3), 96.4% (1.0), 95.2% (1.5), 94.0% (9.8), 95.2% (0.8), and 92.4% (4.7) for 3DPM.

Statistical analysis comparing the median concordance rates among tooth positions showed significant differences between positions 16, 14, 26, and 12 for IMPM, between positions 16, 14, 12, 22, 24, and 26 for IOSM-6, and between positions 16, 14, and 26 for 3DPM (*p* < 0.05).

### Evaluation of color mapping images

**Fig. 9:**
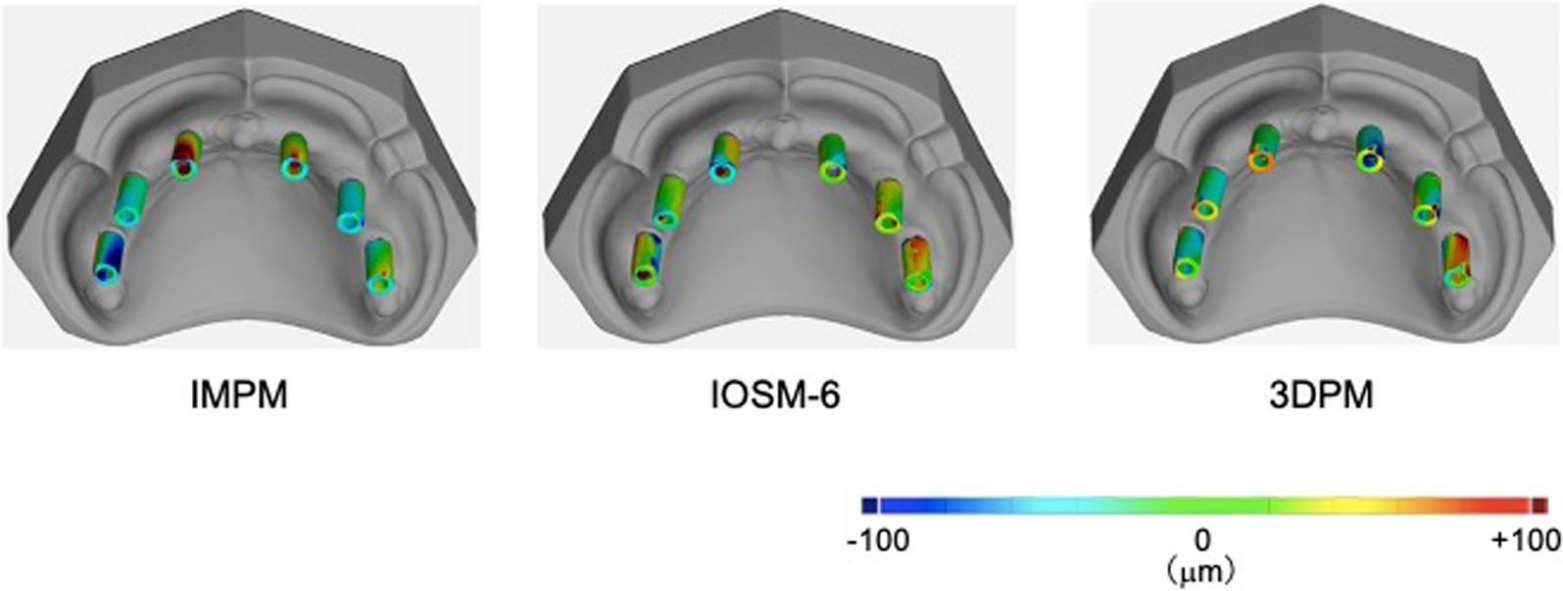
Color mapping images of the working models fabricated using the silicone impression technique, optical impression taken using an intraoral scanner, and working model fabricated using an intraoral scanner and a 3D printer

The color mapping images of the best-fit data obtained for IMPM, IOSM-6, and 3DPM are shown in Fig. 9.

The color mapping image of IMPM showed negative displacement in the upper part of all scanbodies, while a large positive displacement was observed in the scanbodies at positions 12 and 22. In IOSM-6, most scanbodies showed displacement of less than 20 μm, except for the buccal scanbody at position 26, which showed a positive displacement. For 3DPM, the scanbody at position 26 showed a similar pattern to that shown for IOSM-6, while negative and positive displacements were observed in the upper part of the scanbody at position 22 and 12, respectively.

## Discussion

Currently, implant superstructures are fabricated by obtaining the impression of implants using a silicone impression material, followed by pouring plaster into the impression. This process requires impression materials, impression copings, implant analogs, and working models of the implants; therefore, there are problems associated with this process, such as the disposal of infectious waste, increased risk of infection, and increased technical complexity. In this study, to overcome these problems, we used an intraoral scanner, a digital dental technology, to obtain optical impressions of various scanning areas of the master model, fabricated working models of implants using a 3D printer utilizing the scanning data of six scanbodies, and compared the implant position reproducibility of the models with that of the working models fabricated using the conventional silicone impression material.

We investigated the concordance rate for each scanbody region using the optical impression technique with an intraoral scanner and found that IOSM-1 and IOSM-2 showed significantly higher median concordance rates than IOSM-3, IOSM-4, IOSM-5, and IOSM-6, with a greater interquartile range as the size of the scanning area increased. These findings suggest that in the optical impression technique using an intraoral scanner, which constructs 3D data by continuously acquiring scanned data, a larger scanning area and a greater amount of resulting data correlated with a larger error and a lower implant position reproducibility. The scanning trajectories of IOSM-1 and IOSM-2 were close to straight lines, whereas those of IOSM-3, IOSM-4, IOSM-5, and IOSM-6 were a combination of straight and curved lines, suggesting that the accuracy of impressions with an intraoral scanner is affected when the scanning trajectory includes curved lines. However, no statistically significant difference was observed between IOSM-1 and IOSM-2, indicating that higher impression accuracy may be expected in implant-supported fixed dental prostheses for molars with up to three units.

The mean concordance rates of the six scanbodies of IOSM-6 and 3DPM were significantly higher than those of IMPM, demonstrating that the impression accuracy of the intraoral scanner and the modeling accuracy of the 3D printer were superior to those of the models fabricated using the conventional silicone impression technique. Natsubori et al., Miyoshi et al., and Fukazawa et al. implanted multiple implants in an edentulous model, attached abutments to the implants, and then used an intraoral scanner to scan the alveolar ridge and abutments simultaneously to verify the accuracy of the intraoral scanner. They found that longer and larger area scanning by intraoral scanners led to greater impression accuracy errors and concluded that digital scanning by intraoral scanners should be applied to cases with a small number of implants [29–31]. On the contrary, in this study, we used the implant optical impression technique recommended by the manufacturer. After scanning the alveolar ridge of the master model (the first scanning), the scanbody attached to the implant was scanned (the second scanning), and the scanning data were superimposed on the attached personal computer for verification. The results showed that assuming a full-arch implant case, the optical impression technique of the intraoral scanner could show high implant position reproducibility in a jaw model. These results were contrary to the reports of Natsubori et al., Miyoshi et al., and Fukazawa et al. [29–31]. However, this is probably because the alveolar ridge was scanned before the scanbody. This made the alveolar ridge scanning data a landmark for the scanbody to be added later and clarified the positional relationship of the scanbody. Additionally, in 2018, Alshawaf et al. compared the precision of working models by mimicking jaws with a few missing teeth fabricated using conventional impression materials with those fabricated using an intraoral scanner and a 3D printer; they found a substantially lower precision for models fabricated using an intraoral scanner and a 3D printer than for models fabricated using conventional impression materials [32]. The discrepancy between the two studies may be attributed to the differences in the precision of the intraoral scanners and 3D printers used and the angle at which the 3D printer formed the working models. In this study, the Trios3 intraoral scanner and the P30 3D printer were used, which have been proven to be highly precise and superior to other products [13–15, 33, 34]. Tamaki et al. fabricated maxillary dentures using a 3D printer at 0°, 45°, and 90° and found that the maxillary denture fabricated at 45° showed the highest precision [35]. Burde et al. and Camardella et al. reported that when fabricating a 3D-printed model of a horseshoe-shaped dental arch, providing bars connecting to the palate in the bilateral molar regions prevented dimensional deformation of the 3D-printed model [36, 37]. Based on the results obtained in the present study, we believe that providing multiple support materials when forming models on a 3D printer reduces the dimensional deformation of 3D-printed models. These factors may have led to the improved impression precision of the intraoral scanner and modeling precision of the 3D printer, resulting in the superior implant position reproducibility of IOSM-6 and 3DPM. In contrast, the IMPM showed the lowest concordance rate, possibly due to the permanent strain caused by the removal of the silicone impression material, shrinkage of the silicone impression material, and setting expansion of the plaster.

We compared the scanbody concordance rates at each tooth position. IMPM showed low concordance rates with large IQRs at positions 12 and 22, whereas high concordance rates were observed at positions 16, 14, 24, and 26, where the scanbodies were placed perpendicular to the virtual occlusal plane. Color mapping images showed negative displacement in the upper parts of all the scanbodies. This may be because the permanent strain generated during the removal procedure of the silicone impression material remained in the impression material around the impression copings. A positive displacement of up to 100 μm was observed on the labial side of the upper part of the scanbody at positions 12 and 22. This may be attributed to the fact that the implants at positions 12 and 22 remained inclined and were more easily affected by the removal of the silicone impression material than those at other positions. On the other hand, no significant difference in the concordance rate was observed in between the most distant scanbodies, i.e., those at positions 16 and 26, indicating that IMPM had no effect on implant position reproducibility even when the distance between implants was long. In IOSM-6, the scanbody at position 26 showed a lower concordance rate than that at other positions. Color mapping revealed a positive displacement up to 100 μm in the mesial side of the scanbody at position 26. In addition, since a significant difference in the concordance rate was observed in between the most distant scanbodies at positions 16 and 26, in IOSM-6, the implant position reproducibility was considered to decrease when the distance between implants increases. When an optical impression is taken with an intraoral scanner, the scanning data are stacked to construct the 3D data. Position 26 was both the starting and ending points of scanning; therefore, the scanbody at this position was significantly affected by the error in data stacking, which might have led to the above result. In IOSM-6, the scanbodies at positions 16, 14, 12, 22, and 24 showed high concordance rates and small IQRs, indicating that the inclination of the implants had a slight effect on impression precision. In 3DPM, similar to IOSM-6, the scanbody at position 26 showed a lower concordance rate than that at other positions. The IQR of position 26 of 3DPM was also larger than that of the same position of IOSM-6 and the color mapping image of 3DPM showed a positive displacement of up to 100 μm on the buccal side of the scanbody at position 26. These findings suggest that the error related to the modeling precision of the 3D printer increases when the precision of the optical impression obtained using an intraoral scanner decreases. The scanbody at position 22 on 3DPM showed the largest IQR, and the color mapping image revealed a negative displacement of up to 100 μm on the labial side of the upper part of the scanbody at this position. The volume of the print material was the largest during the forming procedure at this position, and thus was greatly affected by the polymerization shrinkage of the printing material, which may have led to the above results. However, the positive displacement observed at the upper part of the scanbody at position 12 indicates the influence of the polymerization deformation of the printing material. At the same time, the IQR of scanbodies at each position on 3DPM was smaller than that on IMPM, indicating that the working models fabricated using an intraoral scanner and 3D printer had lesser error margin than those fabricated using the conventional silicone impression method.

These results confirm that the optical impression technique using an intraoral scanner provides an impression with higher precision than the conventional silicone impression technique. In addition, the combined use of an intraoral scanner and 3D printer enabled the fabrication of working models with high implant position reproducibility. In this study, tissue level implants were used to facilitate confirmation of the junction status between the implant and scanbody. However, in recent dental practice, bone level implants are often used for this purpose. In addition, a jaw model was used in this experiment. In comparison with a patient’s oral cavity, a jaw model (1) allows better manipulation of the intraoral scanner, (2) is free from the patient’s body motion, (3) is invulnerable to the hindrance of image acquisition by saliva, and (4) has no movable mucosa. Based on these favorable features, a high implant position reproducibility of optical impressions obtained using an intraoral scanner can be expected. At the same time, one should bear in mind that the depth of implant insertion and the experience level of operators may affect the accuracy of the impression. Therefore, in the future, we intend to use bone level implants in a patient dummy with an edentulous ridge to verify the influence of implant depth and operator experience on impression accuracy. Additionally, the best-fit algorithm allowed intuitive visualization of the amount and direction of displacement of the scanbody by color mapping in this study. However, Sanda et al. reported that large scanning data may cause errors in the actual positional relationship between the reference and test data, possibly causing an underestimation of errors between images [38]. Thus, in the future, we intend to evaluate errors in the distance and angle of the scanbody, which are considered to be more reliable.

## Conclusions

In this in vitro study, we examined the effect of the size of the intraoral scanning area on implant position reproducibility and compared the implant position reproducibility of plaster models fabricated using the silicone impression technique, digital models obtained using an intraoral scanner, and 3D-printed models fabricated using an intraoral scanner. As a result, although the implant position reproducibility of the optical impression technique using an intraoral scanner decreased with an increase in the scanning area, we found a fair possibility that digital models obtained by the optical impression technique using an intraoral scanner and 3D-printed models fabricated using an intraoral scanner may provide higher implant position reproducibility than plaster models fabricated using the conventional silicone impression technique.

## Data Availability

All data generated or analyzed during this study are included in this published article.

## Abbreviations

3D: Three-dimensional
IMPM: Impression working model
IOSM: Intraoral scanner model
3DPM: 3D-printed models
CAD: Computer aided design
CAM: Computer aided manufacturing
STL: Standard triangulated language
IQR: Interquartile range
